# The regulation of triglyceride and glycogen storage by Glucose transporter 1 ( *Glut1* ) in *Drosophila* fat tissue

**DOI:** 10.17912/micropub.biology.001134

**Published:** 2024-03-01

**Authors:** Louis S. Betz, Justin R. DiAngelo

**Affiliations:** 1 Division of Science, Pennsylvania State University, Berks Campus, Reading, PA, USA

## Abstract

Obesity reflects an imbalance in nutrient storage resulting in excess fat accumulation. The molecules that tissues use to regulate nutrient storage are not well understood. A previously published genetic screen using *Drosophila melanogaster* larvae identified *Glut1* , a transmembrane glucose transporter, as a potential obesity gene. To identify the adipose-specific functions of this gene, *Glut1* levels were decreased using RNAi targeted to fly fat tissue. Adult *Glut1 RNAi* flies have lower glycogen and triglyceride levels, as well as decreased *FASN1* RNA expression. This suggests that *Glut1* functions to promote glycogen and triglyceride storage and fatty acid synthesis in *Drosophila* adipose tissue.

**Figure 1. Glut1 RNAi in Drosophila fat tissue reduces triglyceride and glycogen storage:**
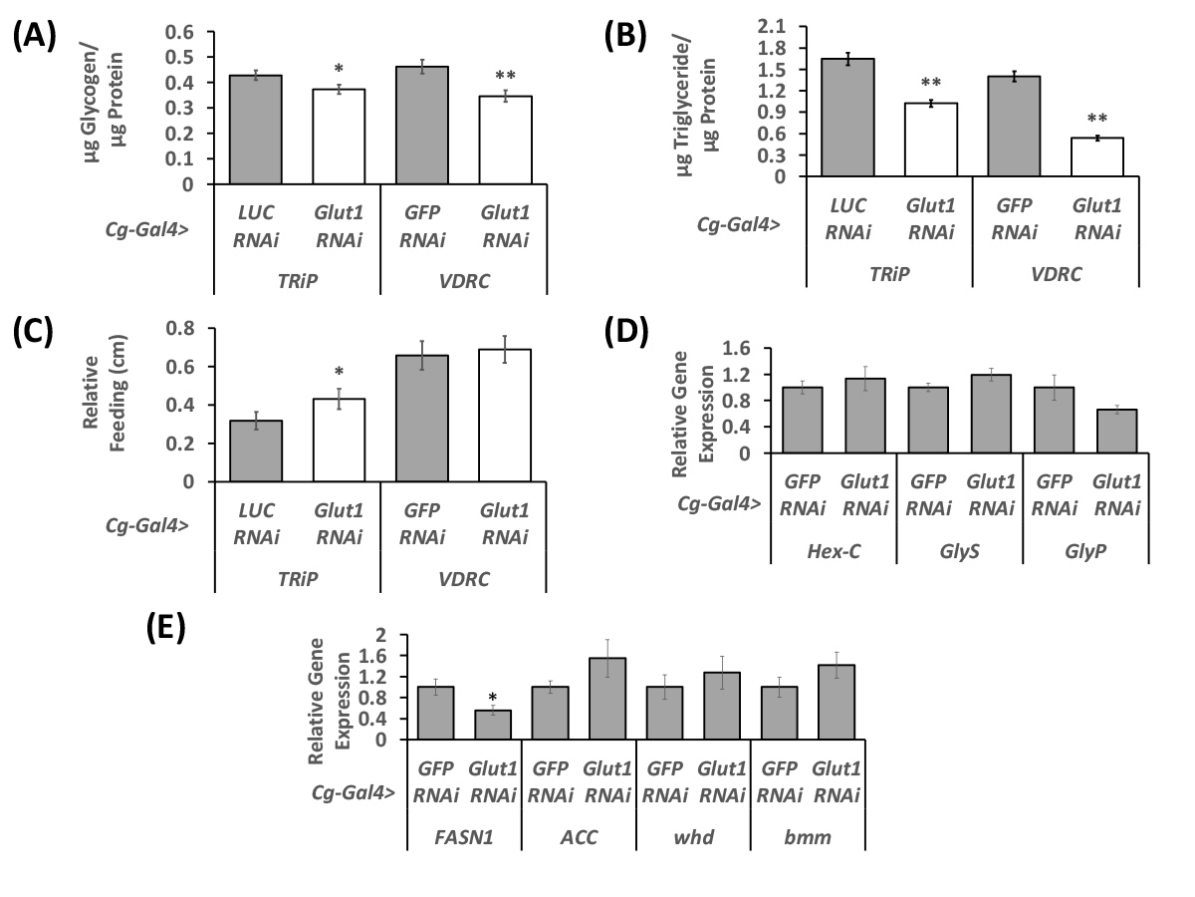
(A) Glycogen normalized to protein, and (B) triglyceride normalized to protein were measured in *Cg-Gal4> Glut1 RNAi-TRiP* and *Cg-Gal4> Glut1 RNAi-VDRC* flies and compared to *Cg-Gal4>LUC RNAi* and *Cg-Gal4>GFP RNAi* controls, respectively (n=20-36). (C) Food consumption after 24 hours was measured in one week old females using a capillary feeding assay. (D-E) qPCR was used to measure the expression of (D) glucose and glycogen metabolism genes (Hexokinase C ( *HexC* ), Glycogen Synthase ( *GlyS* ), Glycogen Phosphorylase *( GlyP* )) and (E) lipid metabolism genes (Fatty acid Synthase 1 ( *FASN1* ), Acetyl CoA Carboxylase ( *ACC* ), withered ( *whd* ), and brummer ( *bmm* )) in cuticles with fat bodies attached dissected from one week old female *Cg-Gal4> Glut1 RNAi-VDRC* flies and compared to *Cg-Gal4>GFP RNAi* controls. Expression levels were measured and divided to *rp49* levels and are displayed with the controls set to 1 and the levels of each gene in the *Glut1 RNAi* normalized relative to the controls (n=11-12). Values are mean ± standard error. **, P < 0.01, *, P<0.05 by two-tailed Student's t test.

## Description

Obesity is a metabolic disease that affects both men and women of all ages on a global scale; nearly one-third of the world population is now classified as overweight or obese (Chooi et al. 2019) . Obesity is the result of an imbalance of energy output and storage within the body causing an excess of stored triglycerides. The study of the mechanisms behind macromolecule storage and use is becoming increasingly necessary as the rate of obesity and its related diseases, type II diabetes and heart disease, all continue to rise (Hruby et al, 2015) . The study of the genes and mechanisms that impact energy homeostasis may be integral to countering the rise in obesity.

The fruit fly, *Drosophila melanogaster* , has been identified as an excellent model organism to study metabolism because metabolic pathways are highly conserved, and fruit flies provide a lens into the overall balance of triglyceride and glycogen storage and usage (Heier et al, 2018; Musselman and Kuhnlein, 2018) . There are a vast number of genes involved in regulating metabolism; however, most of these genes are either not yet known or their functions are not well understood. To identify genes important for triglyceride storage, genetic screens have been performed using the *Drosophila* system. One potential gene important for regulating triglyceride storage identified in a buoyancy screen in *Drosophila* larvae is *Glut1* (Reis et al, 2010) . *Glut1* is a transmembrane glucose transporter and is homologous to human *Glut1 -5* genes (Escher et al, 1999) . *Drosophila* have two glucose transporters, *Glut1* and *Glut3* ; *Glut3* is expressed only in testes, while *Glut1* has a more ubiquitous expression pattern (Chintapalli et al. 2007) . Ubiquitously overexpressing *Glut1* results in glucose uptake *in vivo* in *Drosophila* imaginal discs (Volkenhoff et al. 2018) . In addition, targeting *Glut1* overexpression to neurons can rescue some of the neurodegenerative effects of a fly model of amyloid beta toxicity suggesting that Glut1 is important for neuronal function (Niccoli et al. 2016) . Despite this knowledge of *Glut1* function in neurons *,* the role of *Glut1 ,* and the ramifications of a reduction in *Glut1* in fat tissue has not been explored.

In this study, *Glut1* expression was decreased specifically in the fat body using RNAi and glycogen storage, triglyceride storage, and feeding were characterized. *Glut1* was knocked down in the fat body by combining the fat body specific *Cg-Gal4* line with two distinct *Glut1 RNAi* lines (TRiP and VDRC). Triglycerides, glycogen, and free glucose were measured in whole animals and normalized to total protein content. The glycogen/protein ( Fig. 1A ) and triglyceride/protein ( Fig. 1B ) ratios in fat-body specific *Glut1 RNAi* flies were decreased compared to the appropriate control. This decrease in nutrient storage could be due to a decrease in food consumption in *Glut1 RNAi* flies. To test this hypothesis, feeding was measured over a 24-hour period using a CAFÉ assay. Contrary to this hypothesis, relative food consumption did not decrease in *Glut1 RNAi* flies ( Fig. 1C ). This suggests that the decrease in triglyceride and glycogen storage is not due to altered food consumption; however, since *Cg-Gal4* is expressed during the development it is possible that food consumption could have been altered during the larval stage and further experimentation is necessary to address this possibility.

To further understand the nutrient storage phenotypes observed in *Glut1 RNAi* flies, genes integral for glycogen and triglyceride storage and breakdown were analyzed via qPCR using dissected abdomen cuticles with fat bodies attached. The genes integral for glycogen metabolism analyzed were the glycolytic enzyme hexokinase C ( *HexC* ), the glycogen synthesis enzyme glycogen synthase ( *GlyS ),* and the glycogen breakdown enzyme glycogen phosphorylase ( *GlyP* ). There were no changes in the levels of any of these glycogen regulating genes (Fig 1D), suggesting that the decrease in glycogen storage in *Glut1 RNAi* flies is not due to changes in the expression of these genes. It is possible that this glycogen phenotype may be due to less glucose entering the cell via *Glut1* ; it is also possible that there are non-transcriptional changes in these glycogen regulating genes. Additional experiments are necessary to distinguish between these possibilities.

Genes that regulate triglyceride storage were also analyzed. We measured the expression of acetyl-CoA carboxylase ( *ACC* ) and fatty acid synthase 1 ( *FASN1* ), two enzymes important for the synthesis of fatty acids *.* We also measured the expression of the triglyceride lipase brummer ( *bmm)* and withered *(whd* ), the *Drosophila* homologue of carnitine acyltransferase 1 ( *CPT1)* which is integral to escorting free fatty acids into the mitochondria to be used for beta-oxidation *.* The levels of *ACC ,* *bmm* and *whd* were similar in *Glut1 RNAi* flies and controls; however, *FASN1* was decreased in *Glut1 RNAi* flies (Fig 1E). This decrease in *FASN1* expression suggests that less fatty acids are being made when *Glut1* is knocked down and could contribute to the decrease in triglyceride storage in these flies.

Despite the lack of knowledge about the function of *Glut1* to regulate *Drosophila* metabolism, the functions of glucose transporters in mammals are better characterized *.* Homozygous *Glut1* knockout mice are embryonic lethal and *Glut1* heterozygotes have decreased brain glucose uptake (Heilig et al., 2003; Wang et al., 2006) , but the function of Glut1 in mammalian adipose tissue is not well understood. However, the function of another glucose transporter, Glut4, has been studied in mammalian fat tissue. Adipose-specific *Glut4* knockout mice have significant reductions in glucose uptake, glycolysis, and glycogen synthesis (Abel et al. 2001) . We show here that this decrease in glycogen synthesis is conserved from *Drosophila* to mice, potentially due to less glucose uptake and thus less substrate to form glycogen. Interestingly, adipose-specific *Glut4* knockout mice have no change in fat pad size or weight, suggesting little change in triglyceride storage (Abel et al., 2001) . However, triglyceride levels in isolated adipose tissue were not measured, so it is possible that triglyceride storage is altered in these mice and this triglyceride storage phenotype may also be conserved from flies to mammals.

The genetic screen that identified *Glut1* as a potential anti-obesity gene in *Drosophila* showed that *Glut1* mutants have increased buoyancy, suggesting that loss of *Glut1* increases triglyceride storage (Reis et al., 2010) . This contrasts with the results shown here that inducing RNAi towards *Glut1* specifically in fat tissue results in less triglycerides. It is possible that Glut1 acts in multiple tissues and loss of *Glut1* throughout the entire animal in the *Glut1* mutants used in the buoyancy screen may alter organismal lipid storage differently compared to when *Glut1* expression is decreased only in fat tissue. Another possibility is that *Glut1* functions differently in fat tissue in larvae and adult flies. A recent study has shown that male larvae store more triglycerides than female larvae which is the opposite of the sexual dimorphism seen in fat storage in adult flies (Diaz et al., 2023) . Therefore, it is possible that the apparent contrasting triglyceride storage phenotypes in *Glut1* mutant larvae and adult flies where *Glut1* is decreased specifically in fat tissue may be due to underlying differences in lipid metabolism in fat cells in larvae and adult flies. Future experimentation is necessary to address these possibilities.

Previous studies have shown that *Drosophila* larvae upregulate the levels of *FASN1* mRNA in response to being fed a high sugar diet (Musselman et al., 2013; Sassu et al. 2012; Zinke et al., 2002) . Therefore, it is possible that altering sugar levels in *Glut1 RNAi* fat cells could regulate the levels of *FASN1* mRNA and potentially lead to the blunted triglyceride storage observed in these flies. However, the mechanism of how decreasing *Glut1* leads to both decreased triglyceride storage and *FASN1* levels needs to be elucidated. In addition, whether other enzymes important for the storage of glycogen or triglycerides are altered in *Glut1 RNAi* fat cells and whether Glut1 functions in other metabolic tissues such as muscle and intestine to regulate metabolism in flies are still open questions. Together, the results of this study expand our knowledge of the genes important for regulating the storage of nutrients in *Drosophila* and further our understanding of the role of glucose transport in fat tissue metabolic function.

## Methods

**Fly Genetics** - Virgin GAL4 females were crossed to UAS males and crosses were grown at 25°C on a 12h:12h light: dark cycle. Flies were grown on standard sugar-cornmeal-yeast medium (9 g *Drosophila* agar (Genesee Scientific), 100 mL Karo Lite Corn Syrup, 65 g cornmeal, 40 g sucrose, and 25 g whole yeast in 1.25 L water).

**Triglyceride, Glycogen, and Protein Measurements** - One-week-old female flies were used throughout this study. Two whole flies were homogenized in lysis buffer (140 mM NaCl, 50 mM Tris-HCl, pH 7.5, 0.1% Triton-X, and 1X protease inhibitor (ThermoFisher, Waltham, MA, USA)). Triglyceride, glycogen, and protein were measured using the Infinity Triglyceride Reagent kit (ThermoFisher, Waltham, MA, USA), Glucose Oxidase reagent kit (ThermoFisher, Waltham, MA, USA), and Pierce BCA Protein Assay kit (ThermoFisher, Waltham, MA, USA), respectively, accordingly to manufacturer's instructions and as described previously (Bennick et al., 2019) .

**CAFÉ Assay** - Food consumption was measured using the capillary feeding (CAFÉ) assay (Ja et al., 2007) . Briefly, three 1-week-old female flies were housed in a vial with 1% agar as a water source. Flies were fed with a dyed 5% sucrose in a Drummond 5 μL capillary tube (ThermoFisher, Waltham, MA, USA) capped with mineral oil, as their sole food source. Flies were kept at 25 ºC and the amount of sucrose consumed was measured after 24 h. Vials without flies were used to account for any sucrose evaporation.

**Gene Expression Analysis** - Abdomen cuticles with fat bodies attached were dissected from 15 one-week-old female flies and were homogenized in TRIzol RNA Extraction Reagent (Invitrogen, Waltham, MA, USA) according to manufacturer's instructions. Five μg of extracted RNA samples was DNase treated using DNA- *free* DNA Removal Kit (Invitrogen, Waltham, MA, USA). Then, 0.25 μg of DNase treated RNA was reverse transcribed using qScript Ultra Supermix (QuantaBio, Gaithersburg, MD, USA) according to manufacturer's instructions. qPCR reactions were generated using 1 μL of cDNA, 200 nM primers, and 1 × Perfecta SYBR Green (Quanta Biosciences, Gaithersburg, MD, USA) in a 25 μL reaction. qPCR was performed on a StepOnePlus instrument using the following cycling conditions: initial denaturation at 95°C for 3 min, 40 cycles of 30 s at 95°C, 60 s at 60°C and 30 s at 72°C followed by a standard melt curve. Expression of all genes were normalized to *rp49* . Primer sequences used were:

*HexC* (For 5′-TCGAGGCGGTTACAACTAAGA-3′ and Rev 5′-ACGTACGTGGGAAAACACTTG-3′), *GlyS* (For 5′-CGCGAGGCTATAAAATCCAC-3′ and Rev 5′- GGCAATCATAAAGCCAAGGA-3′), *GlyP* (For 5′-AACTTCCAGCGCAATGTAGC-3′ and Rev 5′-TGGGATCCTTCTTGATCCTG-3′), *FASN1* (For 5′-CTGGCTGAGCAAGATTGTGTG-3′ and Rev 5′-TCGCACAACCAGAGCGTAGTA), *ACC* (For 5′- AGATGCAGAACGATGTCCGC -3′ and Rev5′- CTCTTTGTGAAGCAGCTCCG -3′), *bmm* (For 5′-ACGTGATCATCTCGGAGTTTG-3′ and Rev 5′-ATGGTGTTCTCGTCCAGAATG-3′), *whd* (For 5′-GCCAATGTGATTTCCCTGCTTC-3′ and Rev 5′-CTTTGCCCTTCAGGATTTCCTC-3′) and *rp49* (For 5′-GACGCTTCAAGGGACAGTATCTG-3′ and Rev 5′-AAACGCGGTTCTGCATGAG-3′).

**Statistics** - The results were expressed as mean ± standard error (SE) and average values were compared between *Glut1 RNAi* and the appropriate control flies using a two-tailed Student's t-test as calculated in Microsoft Excel. p < 0.05 was used as the cut off for statistical significance.

## Reagents

**Table:** 

**Strain**	**Genotype**	**Available from**
Cg-Gal4	*w[1118]; P{w[+mC]=Cg-GAL4.A}2*	Bloomington Stock Center #7011
UAS-GFP RNAi	*w1118; UAS-GFP RNAi*	Bloomington Stock Center #9330
UAS-Glut1 RNAi-VDRC	* w1118; UAS- Glut1 RNAi *	VDRC #13326
UAS-Glut1 RNAi-TRiP	*y[1] v[1]; P{y[+t7.7] v[+t1.8]=TRiP.JF03060}attP2*	Bloomington Stock Center #28645
UAS-LUC RNAi	*y[1] v[1]; P{y[+t7.7] v[+t1.8]=TRiP.JF01355}attP2*	Bloomington Stock Center #31603
